# Collaboration strategies for bridging health, behavioral health, and social services in California's Medi‐Cal Whole Person Care Pilot Program

**DOI:** 10.1111/1475-6773.14417

**Published:** 2024-12-04

**Authors:** Emmeline Chuang, Rachel Ross, Nadia Safaeinili, Leigh Ann Haley, Brenna O'Masta, Nadereh Pourat

**Affiliations:** ^1^ School of Social Welfare University of California Berkeley Berkeley California USA; ^2^ School of Medicine Stanford University Stanford California USA; ^3^ Center for Health Policy Research University of California Los Angeles Los Angeles California USA

**Keywords:** integration, Medicaid, qualitative research, social care, social determinants of health

## Abstract

**Objective:**

To identify collaboration strategies used to integrate health, behavioral health, and social services for Medicaid members in California's Medi‐Cal Whole Person Care Pilot program (WPC).

**Data Sources and Study Setting:**

WPC was a social care intervention implemented to identify and address eligible members' health, behavioral health, and social needs. Data included semi‐structured key informant interviews conducted in 2018–2019 (*n* = 221) and 2021 (*n* = 167); pilot‐level surveys; whole‐network surveys of 507 organizations in all 25 pilots participating in WPC; and documents submitted by pilots to the state. Pilots served a total of 247,887 unique members between 2017 and 2021, the majority of whom were non‐white (72%) and over half of whom experienced homelessness.

**Study Design/Data Collection:**

Data were collected as part of the statewide evaluation of WPC. We analyzed qualitative data to examine strategies used by pilots to integrate care, network data to identify pilots that improved cross‐sector collaboration (i.e., strengthened density or multiplexity of cross‐sector ties) following WPC implementation, and comparative case analysis to identify strategies that differentiated pilots that improved collaboration from those that did not.

**Principal Findings:**

Pilots used multiple strategies to facilitate the integration of care. Network analyses identified 10 pilots that significantly improved either density or multiplexity of cross‐sector ties, and one pilot with high cross‐sector collaboration prior to WPC. Compared to pilots that did not improve cross‐sector collaboration, these pilots meaningfully engaged partners in program design and implementation, used braided funds, and leveraged WPC to support broader systems change. These pilots also reported fewer challenges in developing and managing contractual relationships and ensuring meaningful use of data‐sharing infrastructure by frontline staff responsible for care coordination.

**Conclusions:**

Data sharing is necessary but not sufficient for systems alignment. Collaboration strategies focused on addressing financial barriers to integration and strengthening normative and interpersonal integration are also needed.

**What is known on this topic**

- Social care interventions that address patients' health‐related social needs can improve access, quality, and outcomes of care for individuals with complex medical and social needs.
- Social care interventions often require health care organizations to collaborate with human services partners to ensure services are integrated.
- Differences in mission, professional roles, and modes for distributing resources challenge health and human services integration efforts.

**What this study adds**

- We use data from the statewide evaluation of a social care intervention to identify collaboration strategies used to integrate health, behavioral health, and social services for eligible Medicaid members.
- Collaboration strategies are perceived as most important for aligning operations and services focused on strengthening physical, legal, or financial ties or shared mission, vision, and understanding between cross‐sector partners.
- Data‐sharing infrastructure and agreements were necessary but not sufficient for cross‐sector collaboration in the absence of other approaches (e.g., collaborative governance and braided funding).

## 1 INTRODUCTION

Growing awareness of the impact of social risk factors on health has heightened pressure for health care organizations and payers to address clients' health‐related social needs (HRSN), for example, for transportation, housing, or nutrition supports.^1^, ^2^ In the US, current approaches to addressing HRSN in clinical settings occur primarily at the individual level, through screening and referral of clients or provision of care coordination or care management.^3^ While important, in many communities, screening, referral, and other individual‐level interventions may not improve health unless implemented in tandem with efforts to improve local social services capacity or otherwise change underlying systems of care.^4^, ^5^, ^6^ Acknowledging this tension, some hospitals and health systems are also engaged in more “upstream” approaches for addressing HRSN, such as supporting construction of affordable housing or developing food pharmacies.^7^, ^8^, ^9^

Health care organizations' efforts to address HRSN at either the individual or community level typically require at least some collaboration with human service organizations and other partners.^2^, ^3^, ^10^ Such collaboration has been described as essential for improving population health and well‐being,^11^, ^12^ but difficult to achieve due to the inclusion of partners with potentially different priorities, regulations, funding, staffing, and capacity.^13^, ^14^ Currently, relatively little is known about factors influencing the success of cross‐sector system alignment efforts, particularly once financial barriers to collaboration are reduced.

This study draws on mixed methods data from a statewide demonstration program, California's Medi‐Cal Whole Person Care (WPC) pilot program, to identify collaboration strategies used to integrate health, behavioral health, and social services for Medicaid members. Heterogeneity in WPC pilots' approaches provide a unique opportunity to identify strategies used by pilots to align services and operations across different sectors of care. We also examine strategies associated with improved cross‐sector collaboration following WPC implementation.

### 1.1 Medi‐Cal Whole Person Care Pilot Program

WPC was a $3 billion social care intervention implemented as part of California's Medicaid Section 1115(a) waiver demonstration. Under WPC, 25 pilots developed infrastructure and services needed to promote integrated delivery of care for Medicaid members using acute services in multiple service sectors, with the aim of improving member health and well‐being. Per state requirements, all pilots were implemented by cross‐sector partnerships that included county agencies, community‐based organizations, and at least one Medicaid managed care plan; each pilot also designated a single lead entity responsible for fiscal administration and reporting.

Pilots were required to serve one or more “populations of focus,” but otherwise tailored their programs to reflect local needs and resources. WPC‐eligible populations included Medicaid members who were high utilizers of acute health care services; experiencing or at‐risk of homelessness; affected by serious mental illness or substance use disorders; recently released from jail or prison; or with multiple chronic conditions. All pilots offered care coordination and at least some housing assistance, benefits assistance, and transportation assistance; the inclusion of other services such as medical respite, sobering centers, or re‐entry services varied. All pilots also developed new infrastructure to improve the integration of care in their communities. (See Appendix S1 for additional information).

Between 2017 and 2021, pilots served a cumulative total of 247,887 unique members, over half of whom were Hispanic, Latino, Black, or African American (54%) and 28% of whom were White. Over half of the enrolled members experienced homelessness (53%), close to a quarter had serious mental illness and/or substance use disorders (24%), and a quarter were justice‐involved (25%). Statewide evaluation results suggest that, overall, WPC increased member access to substance abuse treatment and specialty care services and reduced emergency department use, hospitalizations, and overall cost of care.^15^ However, pilots varied considerably in program design and implementation,^16^, ^17^ including collaboration strategies used to improve integration of care,^15^ providing a unique opportunity to identify common lessons learned and determine which strategies may have been most effective.

### 1.2 Conceptual framework

**FIGURE 1:**
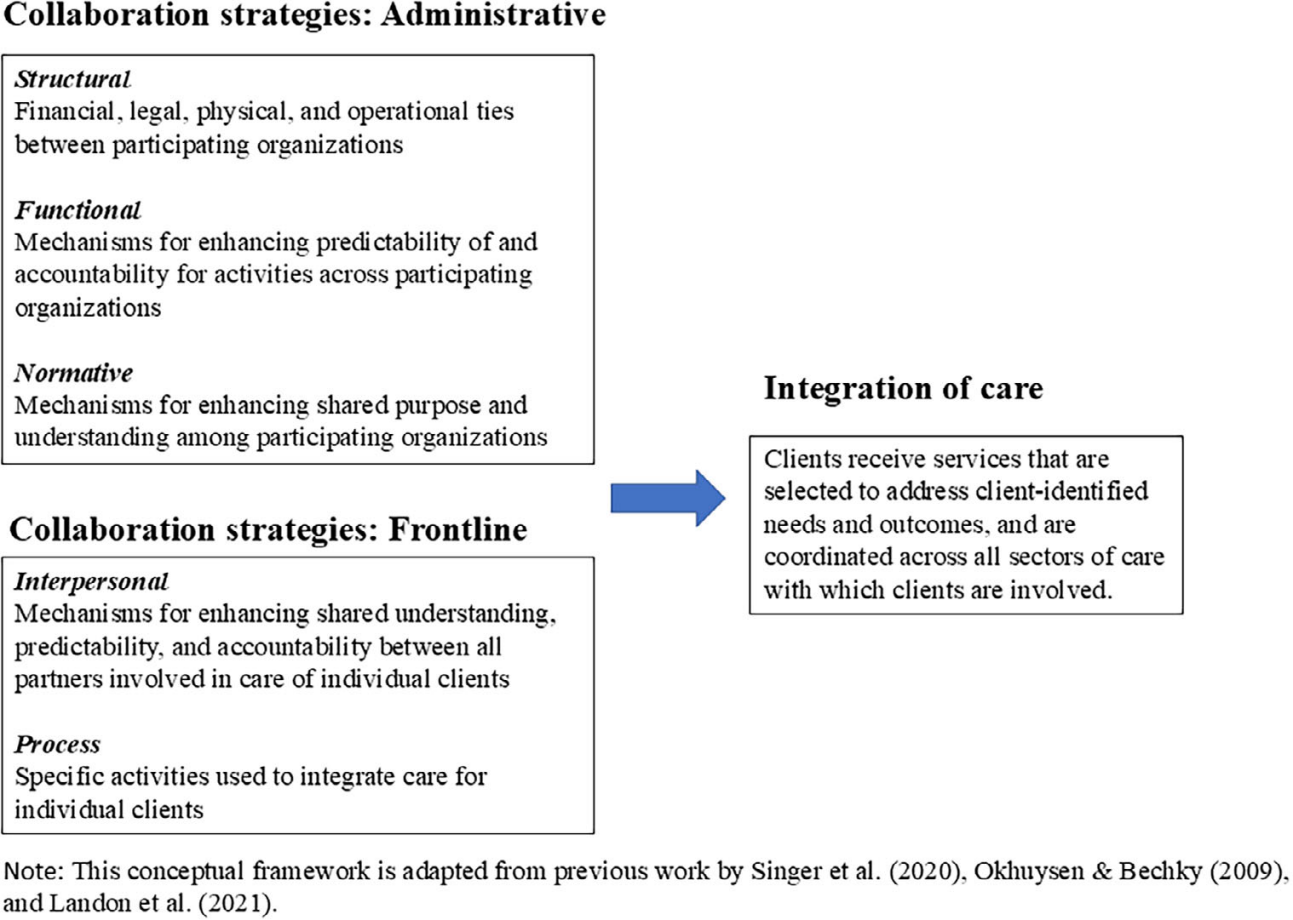
Conceptual framework of collaboration strategies influencing integration of care.

Integration of care occurs when “clients receive services that address client‐identified needs and are coordinated across all sectors with which clients are involved.”^18^, ^19^ Our study of collaboration strategies used to integrate care is guided by the conceptual framework in Figure 1. This framework is informed by prior research on care integration, particularly the comprehensive theory of care integration^20^ and the cross‐sector alignment framework.^21^

The comprehensive theory of care integration describes integration as multidimensional and multilevel, and as influenced by *organizational features*, *social features*, and *process activities*. Organizational features reflect physical, legal, operational, and financial ties between organizations or teams within organizations (*structural integration*) as well as formal policies or protocols for coordinating activities and supporting decision‐making and accountability between these entities (*functional integration*). Social features encompass shared mission, vision, culture (*normative integration*) and quality of collaboration among staff, caregivers, and clients (*interpersonal integration*). Finally, *process integration* refers to specific actions or activities taken to ensure coordination of care for individual clients.

Extant research applying this theory has confirmed the importance of care integration for provider/staff experience and quality of care,^22^, ^23^ but has primarily focused on care integration within or between health systems rather than across different sectors of care, for example, health and human services.^22^, ^24^ Thus, the cross‐sector alignment framework complements the theory of care integration by highlighting the specific importance of “partnership‐level” strategies such as governance practices, shared data and measurement systems, and financial incentives for integrating care across sectors.^21^ Collectively, these two frameworks provide a comprehensive conceptual model for identifying and organizing collaboration strategies used by WPC pilots to improve care integration at multiple levels.

## 2 METHODS

We used a multiple case study design,^25^ with pilots as the unit of analysis. First, we concurrently analyzed qualitative and survey data to identify collaboration strategies used by each pilot, and categorized each strategy based on the dimension of care integration being influenced (structural, functional, normative, interpersonal, process). Next, we analyzed data from network surveys of WPC partners to identify pilots with increased cross‐sector collaboration following WPC implementation. Finally, we analyzed pilot‐level data to identify collaboration strategies that differentiated pilots that improved cross‐sector collaboration from those that did not.

### 2.1 Data sources and sample

Our sample included all WPC pilots. Each pilot typically served a single county; however, one pilot was comprised of two rural counties that collaborated on financial claiming and data reporting but developed separate partnerships and programs. In our analyses, we treated these two counties as distinct cases, resulting in a final sample of 26. Data were drawn from multiple sources gathered as part of the statewide evaluation of WPC.^15^

#### 2.1.1 Collaboration strategies

Collaboration strategies refer to strategies used to align operations and services across sectors of care.^26^ To identify collaboration strategies used in WPC, we relied primarily on key informant interviews conducted September 2018–May 2019 (*n* = 221 participants) and June–August 2021 (*n* = 167 participants). Key informants included organizational leaders and program managers from different WPC participating entities and frontline supervisors and staff involved in WPC implementation. Interviews took place in person or by telephone using a semi‐structured interview guide. Interview questions were tailored to key informant roles and provided additional insight into strategies used to integrate care. At least two research team members were present at each interview. With key informants' consent, interviews were recorded and professionally transcribed.

Interview transcripts were supplemented and triangulated with data from additional sources. We used applications and biannual narrative reports submitted to the California Department of Health Care Services to obtain additional information on pilots' governance practices and changes in infrastructure and care coordination activities over time, respectively. We used pilot‐level surveys administered to lead entities in 2018, 2020, and 2021 (response rates 96%–100%) to obtain structured data on collaboration strategies and services provided, and quarterly enrollment and utilization reports and invoices to identify the number of unique individuals enrolled and to track WPC service use and expenditures. Detailed information on data sources used to assess each collaboration strategy is available in Appendix S2.

In the statewide evaluation, qualitative data were coded using template analysis,^27^ in which a preliminary codebook of factors hypothesized to affect WPC implementation and impact was refined to incorporate emergent themes. For this study, we selected nine codes relevant to our study aims and aligned with our conceptual framework (e.g., governance, contracting practices, data‐sharing infrastructure, staffing) and recoded content in these domains using the framework in Figure 1, to identify collaboration strategies used to facilitate the integration of care. All qualitative data were coded by at least two research team members; discrepancies in coding were discussed until consensus was reached. Coded data were used to generate case studies summarizing general themes within each code for each pilot. Codes representing each collaboration strategy were then organized by type of integration influenced (e.g., structural, functional, and normative). Salient data from pilot‐level surveys were descriptively analyzed to provide supplemental, structured information on identified collaboration strategies. Qualitative analyses were conducted using NVivo 14.0.^28^ Surveys were analyzed using Stata 16.1.^29^

#### 2.1.2 Improved cross‐sector collaboration

We used network surveys to identify changes in cross‐sector collaboration following WPC implementation. First, we used lists provided by lead entities to develop rosters of WPC‐participating entities and their sector affiliation (physical health, mental health/substance use, social services, justice, or other). Then, we disseminated surveys in which respondents were provided with these rosters and asked about their organization's relationships with each partner. Surveys assessed the following types of ties^30^, ^31^: no relationship; joint advocacy or other joint planning; data sharing (e.g., for needs assessment or care); referrals; communication about member needs or care; and joint service delivery. Ties were assessed at two‐time points: prior to WPC (baseline) and mid‐implementation (2018). Data were symmetrized, meaning we considered the tie present if reported by one partner. A manual review of data from five pilots did not identify any instances of asymmetric responses from participating organizations, meaning if one organization reported a tie as present, the other organization always did too.

We used two network‐based measures to examine changes in cross‐sector collaboration following WPC: *network density* and *multiplexity of ties*. *Network density* describes the proportion of possible relationships within a network that are actually present,^32^ with higher density implying a higher degree of connectedness between partners. In this study, density was operationalized as the proportion of all possible cross‐sector partnership pairs in each pilot that reported ties with each other (out of all possible ties). We focused primarily on the presence of any tie, but also calculated the density of specific types of ties.

*Multiplexity* assesses the extent to which two entities in a network are linked by more than one type of relationship, and is an indicator of tie strength, or quality of inter‐organizational collaboration between organizations.^33^, ^34^, ^35^ We operationalized multiplexity at the pair‐wise level as a count variable (range 0–5) of the number of different ways cross‐sector partners worked together, which we averaged across all pair‐wise relationships in each pilot to reflect the overall multiplexity in the network. We used paired t‐tests to compare changes in density and multiplexity of cross‐sector ties after WPC, overall and for each pilot. Network data were analyzed using UCINET 6.720^36^ and Stata 16.1.^29^

We identified pilots as strengthening cross‐sector collaboration if they significantly increased either density or multiplexity of cross‐sector ties, and as not improving collaboration if they did not increase either one. Case studies were then reviewed to identify collaboration strategies and other organizational or contextual factors (e.g., urbanicity, populations of focus, pre‐existing data‐sharing infrastructure) that differentiated pilots that strengthened cross‐sector collaboration from those that did not. Study activities were approved by the California Committee for the Protection of Human Subjects. [Correction added on 12 December 2024, after first online publication: the phrase “(IRB name redacted for peer review)” has been changed to “California Committee for the Protection of Human Subjects” in the preceding sentence.]

## 3 RESULTS

### 3.1 Partnership characteristics

Over half of the pilots were led by entities embedded within integrated county health and human services agencies (52%; see Table 1); there were led by county behavioral health, and three were led by county human services. In 2018, pilots included a total of 507 organizational partners (average 20 per pilot, range 9–51) from diverse settings. All pilots included Medicaid‐managed care plans and medical providers such as hospitals or federally qualified health centers as partners. All pilots also included at least one human services partner, though the types of partners varied significantly (Table 1). For example, the majority of pilots (80%) included county units responsible for benefits eligibility, but less than a quarter (24%) included child welfare or other child and family services providers.

**TABLE 1:** Characteristics of California Medi‐Cal Whole Person Care (WPC) Pilots (*n* = 26).

	Mean or %	Minimum	Maximum
Pilot characteristics^a^			
Led by county health and human services agency	52%	0	100%
Number of partners (2020)	20	9	51
Types of human service partners included in integration efforts			
Benefits eligibility	80%	0	100%
Children and family services (e.g., child welfare, childcare)	24%	0	100%
Employment and training services	40%	0	100%
Financial assistance (e.g., TANF)	68%	0	100%
In‐home supportive services (IHSS)^b^	64%	0	100%
Justice system (e.g., probation, sheriff's office)	68%	0	100%
Nutrition assistance (e.g., SNAP)	56%	0	100%
Public housing (e.g., local housing authority)	60%	0	100%
Employment and training services	40%	0	100%
Services offered^c^			
Legal services	84%	0	100%
Employment assistance	76%	0	100%
Medical respite	72%	0	100%
Sobering centers	56%	0	100%
Re‐entry services	28%	0	100%
Enrollee characteristics			
Number of enrollees (per Pilot)	9975	143	76,107
Mean length of enrollment (months)	14.2	1	60
WPC funds and service expenditures			
Total funds per Pilot (millions)	$123	$5	$1356
% budget spent on services	51%	35%	68%
Mean service expenditures per WPC enrollee	$12,230	$1456	$23,004

Abbreviations: SNAP, Supplemental Nutrition Assistance program; TANF, Temporary Assistance for Needy Families.

^a^ All pilots included at least one Medicaid‐managed care plan and local health care providers such as hospitals or federally qualified health centers as partners.

^b^ IHSS is California's largest Medicaid home and community‐based services program and provides personal care and other services to eligible older or disabled individuals as an alternative to out‐of‐home placement.

^c^ All pilots offered care coordination, housing assistance, benefits assistance, and transportation assistance.

*Source*: Authors' analyses of surveys administered to Pilots as well as of invoices, enrollment reports, and narrative reports submitted by lead entities to the state Medicaid agency July 2016 – December 2021.

### 3.2 Collaboration strategies used to facilitate integration

Collaboration strategies used by pilots to integrate care are organized by the dimension of integration impacted (see Table 2) and briefly summarized below.

**TABLE 2:** Collaboration strategies used to promote integration within California's Medi‐Cal Whole Person Care (WPC) Pilot Program.

Integration type	Collaboration strategies used	Illustrative quote
Structural	Collaborative governance	“We are a mega agency, with social services, public health, and behavioral health… If [WPC] were housed in one of the three, it would become a [division‐specific] project… to avoid that, we got approval from the board of supervisors to create a new division, WPC.”
	Contracts or other interagency agreements	“Contracts are really useful… there is a financial piece you can write in to say, ‘This is the direction we as a system of care we are wanting to go’… for example, we revised [our housing service bundle] to be more specific about… the kind of integrated care we were looking for.”
	Develop new data sharing or delivery system infrastructure	“We built our electronic care management platform from the ground up… to do care planning, assessments, and drive eligibility decisions based on inclusion criteria… for our team members.”
	Staffing practices such as co‐location, or use of multidisciplinary teams or staff with lived experience	“The foundation of our care coordination approach was multidisciplinary teams representing the variety of sectors serving each client with complex needs.”
	Braided funding	“WPC did not have funding to support direct housing costs, so we combined WPC with Housing and Disability Advocacy Program funds in our contracts.”
Functional	Universal client consent forms for release of information	“Medical, housing, jails… everything is under one unique identifier and with our WPC consent, we will have full access to [all of] it.”
	Standardized enrollment and/or referral protocols	“On a typical day we look at the doctor's schedules… When they [eligible members] check in, the staff notify us, and we go ahead and talk to them [about WPC] while they're waiting for the doctor …”
Normative	Regular meetings with cross‐sector leaders focused on joint problem solving, advocacy, and information sharing	“We have a network of partners getting together routinely to discuss the same types of issues and solutions… And that model of bringing everybody together and forcing them to do it routinely and consistently has helped solidify relationships.”
	WPC as part of broader systems change effort	“For us, WPC was an opportunity to bring funding to the [whole] county to… expand things we were already doing, build a more integrated system… and leverage newly developed infrastructure to benefit broader populations not enrolled in WPC.”
Interpersonal	Client‐centered approaches for outreach and engagement	“Being trauma‐informed and using harm reduction is important… being authentic and respectful and not being judgmental, so there's a level of trust built… and patients want to keep coming back.”
	Staff training	“We made a major investment in training… to build everyone's capacity for understanding what care coordination is.”
	Regular case conferences, huddles, or other team meetings involving frontline staff and other partners from different sectors	“We work in a team‐based environment, with multiple staff members working together to meet the entirety of patients' needs. And what supports that is our daily huddle… It brings the team together…gives us a chance to regularly reinforce our internal processes and outcomes, and integrate at the system and team level.”
Process	Comprehensive needs assessment using pre‐specified, structured tool	“We do what's called a comprehensive needs assessment, and the findings of that assessment help in the development of a care plan.”
	Client‐centered care plan accessible to all staff responsible for care coordination	“We keep it client‐centered. Even if to our consternation the goals they've chosen don't quite seem to meet the needs we see they have, we keep them as goals while using motivational interviewing to help them view other goals they may want to consider as well.”
	Regular contact with enrollees (at least 1×/month, preferably in‐person)	“We need to build relationships and prove our worth to patients before asking them to sign [on] to anything… and relationships are built face‐to‐face.”

#### 3.2.1 Structural integration

Pilots used five strategies to facilitate structural integration: collaborative governance, contracts, infrastructure development, staffing, and braided funding.

*Collaborative governance* refers to governing arrangements in which partners are directly engaged in formal decision‐making^37^, ^38^; a central feature is the deliberate inclusion of partners in consensus‐oriented decision‐making, rather than solely in a consultative capacity. While all WPC pilots included partners from different sectors in executive or steering committees and most (85%) included human services partners in planning and implementation, fewer used a collaborative governance approach in which partners from different sectors were consistently included in decision‐making. Pilots that did utilize a collaborative governance approach described it as important for “enhancing relationships” and “creating a shared vision and approach.”

*Contracts or other legally binding agreements* formalizing inter‐agency relationships were described as time‐consuming and complex to develop and manage but necessary for clarifying partner roles and responsibilities and ensuring partner‐level accountability. All pilots developed data‐sharing agreements with at least some partners, and most lead entities contracted out at least some services.

When asked about specific contracting practices, almost all lead entities (92%) reported the use of financial incentives in their contracts with WPC partners. However, the nature and perceived effectiveness of financial incentives varied. Most lead entities incentivized partner participation in learning collaboratives or meetings (69%), data sharing (69%), or achievement of process goals (62%); less than a third (30%) incentivized clinical targets or benchmarks. In general, financial incentives were perceived as effective only when they helped align partner and WPC goals, reinforced partner accountability for fulfilling pre‐identified roles and responsibilities, were compatible with existing billing practices, and were large enough to motivate behavior change.

*Infrastructure development* was a central strategy for integrating care, with all pilots developing at least some new delivery system or data‐sharing infrastructure. New delivery system infrastructure was typically implemented to address gaps in existing systems of care (e.g., medical respite to provide care for members experiencing homelessness who do not require hospitalization but are too ill or frail to be on the streets or in a shelter) or to promote staff proximity (e.g., care navigation centers as “one‐stop shops”). Data‐sharing infrastructure, when developed to support care coordination or other program activities rather than just meet state reporting requirements, was described as beneficial for supporting process integration. For example, 20 pilots implemented electronic care coordination or case management platforms accessible to staff “in the field,” and 17 pilots had systems providing staff with event‐based notifications of emergency department visits or hospitalizations.

Despite successes in developing new infrastructure, many pilots reported difficulty navigating data sharing requirements, particularly related to federal regulations addressing the confidentiality of substance use disorder records,^39^ and frustration with the lack of interoperability of existing electronic health record systems. Once implemented, many pilots also reported challenges with the use of data‐sharing infrastructure by frontline staff, suggesting that infrastructure alone was insufficient for meaningful integration: “Convincing people to use another data system when they have an existing one was challenging… our care coordination platform is only as useful and robust as the people willing to use it and enter information in it.”

#### 3.2.2 Staffing practices

Pilots differed in the type(s) of staff providing care coordination, the settings in which staff were embedded, their proximity and access to staff with other salient expertise, and in staff onboarding and training practices. For example, 13 pilots used a single care coordinator to follow clients across all WPC‐participating settings while other pilots used multiple care coordinators, either as part of a larger care team or embedded within different organizations. Caseloads also varied considerably, ranging from as little as 6 to as high as 100. Specific staffing practices used to improve integration of care included the use of multidisciplinary teams involving staff from health and human service sectors (76%), co‐location of health and human services staff (48%), and cross‐training staff (48%).

#### 3.2.3 Braided funding

Braided funding occurs when multiple, separate funding streams are used to support a common goal, while ensuring funds remain distinct and trackable. In WPC, the use of a 1115(a) waiver allowed pilots to offer services not traditionally covered by Medicaid (“out of the box solutions to client needs”); however, there were still restrictions on how funds could be used. For example, WPC funds could pay for care coordination and housing supports, but not counseling or rent.^3^ To overcome these restrictions, some lead entities (~40%) braided disparate funds as a strategy for providing more integrated care. For example, some lead entities used braided funds to cover staff salaries so staff could simultaneously provide care coordination and direct services. Other lead entities braided funds in contracts with community‐based partners, for example, so that partners could provide clients with care coordination and housing‐related financial assistance, or support the development of new data‐sharing infrastructure.

#### 3.2.4 Functional integration

Pilots used two primary strategies for facilitating functional integration: universal consent forms, and standardized enrollment or referral protocols. *Universal consent forms* securing client consent to share information with other providers was identified by many pilots as essential for improving coordination of care; by 2020, most pilots (62%) reported using universal consent forms to secure client consent for data sharing across WPC partners. Most pilots also reported using *standardized protocols* for enrolling or referring members to WPC (87%) and many (64%) also had protocols for monitoring referral status. These protocols were described as helpful for creating shared understanding among partners, but varied in the extent to which they were consistently followed by frontline staff. Closed loop referrals, that is, outreach after referral to confirm that needs were met, were less common and often identified as a desired area of improvement: “[Partner agency] is really impacted by all the referrals they get, so we have challenges in follow‐up…because they don't have capacity.”

#### 3.2.5 Normative integration

Normative integration reflects the extent to which partners share common mission, vision, and sense of urgency in improving the integration of care. Pilots identified two strategies for strengthening normative integration: *regular meetings with cross‐sector leaders* and *implementing WPC as part of broader systems change efforts*. Regular meetings with cross‐sector leaders were described as important for developing shared understanding of program mission, scope, and other care sectors, and facilitating cross‐agency problem‐solving needed to meaningfully improve normative integration: “To get these partners in the same room once or twice a month sounds so simple, but it was such a game changer… It really changed the culture.”

Pilots also perceived normative integration as stronger when WPC was implemented as part of broader systems change efforts rather than as a stand‐alone program. For example, one pilot used WPC to enhance an existing countywide initiative to improve wraparound services for homeless individuals with serious mental illness. Another pilot used WPC to develop the infrastructure needed to improve the integration of care for all individuals receiving county‐provided services, not just those enrolled in WPC.

#### 3.2.6 Interpersonal integration

At the frontlines of care, pilots identified four primary strategies for enhancing interpersonal integration, or the quality of collaboration between staff and clients: client‐centered outreach and engagement, use of staff with lived experience, staff training, and regular case conferences related to client care. Medically and socially complex individuals eligible for WPC were described as more resistant to seeking and accepting care than “average” Medicaid members.^40^ *Client‐centered approaches for outreach and engagement* such as field‐ or point‐of‐care‐based connections and prioritization of client‐identified rather than program‐directed goals were identified as important for the quality of collaboration with clients. For example, one pilot developed mobile care pods as a way of providing homeless individuals with access to showers, hygiene products, and basic medical care, and “engaging individuals that [otherwise] do not really want to receive services.” All staff described at least some in‐person contact as essential for interpersonal integration. *Use of staff with lived experience* was identified by many pilots as similarly important for client engagement: “The key strategy that helped us was the peer support specialist focused on creating rapport with the homeless population… We saw a huge rise [in client engagement] within the month of bringing him in.”

*Staff training* on topics such as trauma‐informed care and motivational interviewing reinforced the use of client‐centered approaches, and helped staff develop shared understanding of program activities. Finally, *regular case conferences, huddles, or team meetings*, in which partners from different sectors came together to discuss client care and share best practices, were described as important for quality of collaboration between staff (interpersonal integration) and for shared understanding and accountability (process integration). Most pilots (72%) reported use of such meetings to promote accountability for client care, though frequency varied (e.g., daily vs. monthly or “as needed”). One pilot that implemented daily huddles described high‐frequency contact as critical for interpersonal integration.

#### 3.2.7 Process integration

Pilots' strategies for facilitating process integration focused primarily on ensuring accountability and predictability of care coordination services. For example, all pilots required staff to conduct comprehensive needs assessments, often using structured tools to ensure systematic identification of members' needs, develop client‐centered care plans, and meet with members at least once every 30 days to ensure identified needs were being met. Staff reported significant variation in frequency and modality of contact with members, often dictated by caseload, case complexity, and ease of locating members to provide care.

### 3.3 Changes in cross‐sector collaboration

As shown in Table 3, prior to WPC, average network density was 0.51 (range 0.23–0.75), meaning that on average, over half of organizations (51%) had cross‐sector collaborative relationships prior to WPC. The most common type of cross‐sector collaborative relationship between partners was client referrals (mean 0.32), followed by joint planning or advocacy (mean 0.26), and communication about client needs or care (mean 0.25). The least common collaborative relationships were data sharing (mean 0.17) and joint service delivery (mean 0.13). By the end of 2018, approximately 2 years after WPC implementation, average network density increased (mean 0.56; range 0.32–0.81) but changes were not statistically significant (*p* < 0.05). Multiplexity of ties also increased but not in a statistically significant way, from an average of 2.23–2.48, meaning that organizations generally collaborated with cross‐sector partners in two or more ways both before and after WPC. The only cross‐sector collaborative relationships that did increase in a statistically significant way following WPC were joint advocacy or planning, data sharing, and communication about client needs or care.

**TABLE 3:** Changes in cross‐sector collaboration before and after WPC.

	Prior to WPC		After WPC (2018)	
	Mean (SD)	Range	Mean (SD)	Range
Network density: Any tie	0.51 (0.13)	0.23–0.75	0.56 (0.14)	0.32–0.81
Joint advocacy or planning	0.26 (0.13)	0.09–0.58	0.34^a^ (0.15)	0.09–0.75
Data sharing	0.17 (0.12)	0.05–0.44	0.25^a^ (0.14)	0.07–0.61
Client referrals	0.32 (0.12)	0.07–0.59	0.35 (0.13)	0.14–0.67
Communication about client needs or care	0.25 (0.13)	0.08–0.53	0.32^a^ (0.13)	0.16–0.64
Joint service delivery	0.13 (0.11)	0.03–0.46	0.18 (0.13)	0.04–0.53
Multiplexity of ties	2.23 (0.66)	1.39–4.05	2.48 (0.60)	1.35–4.22

*Source*: Authors' analyses of roster‐based network surveys administered to all WPC‐participating entities assessing changes in collaborative relationships before and after WPC implementation. This analysis only includes cross‐sector relationships (i.e., ties between organizations from different sectors).

^a^ Significant change after WPC, assessed using paired *t*‐tests, *p* < 0.05.

Analysis of individual pilot‐level networks indicated that 10 of 26 pilots reported statistically significant increases in either density or multiplexity of cross‐sector ties following WPC. In pilots with statistically significant changes on these measures, average network density increased from 0.45 to 0.61 (16%) and average multiplexity increased from 1.7 to 2.4, meaning organizations collaborated on average in one more way than they had before. One of the remaining pilots had high baseline density (0.75) and multiplexity of ties (4.05), reflecting strong cross‐sector collaboration that increased further after WPC, though not in a statistically significant way; in cross‐case analysis, we categorized this pilot as improving cross‐sector collaboration based on the overall strength of cross‐sector ties, resulting in a total of 11 pilots. Detailed results are available in Appendix S3.

### 3.4 Strategies differentiating pilots that improved cross‐sector collaboration

In cross‐case comparisons, we compared the 11 pilots categorized as improving cross‐sector collaboration to the 15 pilots that did not. Three collaboration strategies differentiated these pilots: collaborative governance, defined in our study as meaningful inclusion of cross‐sector partners in WPC design, planning, and implementation; braiding funds to support the provision of integrated care; and implementing WPC as part of broader cross‐sector systems change efforts (see Table 4). With regards to collaborative governance, all lead entities included WPC partners on executive and steering committees; however, pilots that did not improve cross‐sector collaboration were less inclusive. For example, one pilot included long‐standing community coalition partners in initial program design and implementation, but failed to engage housing or human services partners whose buy‐in and participation were also central to program success. Another pilot only included partners in a consultative capacity, and did not provide them with true decision‐making power, which in turn resulted in lower partner buy‐in. A third pilot used a collaborative governance approach in designing WPC, but not in planning or implementation after funds were awarded. Similarly, with one exception, pilots who braided funds and implemented WPC as part of broader, cross‐sector systems change efforts reported greater successes in integrating care than those who did not. One pilot that did not improve cross‐sector collaboration reported braiding different human services funds (e.g., for benefits eligibility and financial assistance) in hiring county social services staff, but not to improve cross‐sector integration of care. Finally, while we did not identify specific policies and practices that explained these differences, pilots that improved cross‐sector collaboration reported fewer contracting challenges and higher meaningful use of data‐sharing systems by WPC partners; none of the other organizational or contextual factors examined (e.g., urbanicity, populations of focus) differentiated pilots that improved cross‐sector collaboration from those that did not.

**TABLE 4:** Select collaboration strategies used by pilots to facilitate integration of care.

Pilot	Collaborative governance*	Contract incentives^a^	Data sharing^b^	Staffing practices^c^	Braided funding*	Client‐centered engagement^d^	Part of broader cross‐sector systems change*	Did pilot improve cross‐sector collaboration?
1	Yes	a–f	a, b, c	b	Yes	c	Yes	Yes
2	Yes	a, b, c, d, e	a, b, c	b, c	Yes	a, b	Yes	Yes
3	Yes	d	b	b, c	Yes	a, b	Yes	Yes
4	Yes		a	a, b	Yes	a, b, c	Yes	Yes
5	Yes	d	a		Yes	b, c	Yes	Yes
6	Yes	a, b	b, c	a, b, c	Yes	a, b	Yes	Yes
7	Yes	a, d, e	b, c	b	Yes	a, b	Yes	Yes
8	Yes	a, b, c, d, e	b, c	b	Yes	a, b	Yes	Yes
9	Yes	b	a, b, c	a, b	Yes	a, b, c	Yes	Yes
10	Yes	a, c, d	a	a	Yes	a, b	Yes	Yes
11	Yes	a, b	a, b, c	b	Yes	a, b	Yes	Yes
12	No	a, b, c	a, b, c	b, c	No		No	No
13	No	a, b, c, d, e	b, c	a, c	No	a, b	No	No
14	No	a, b, c, d	a, b, c	b, c	No	a, b	No	No
15	No	a, c, d, e, f	c	a, b, c	No	b	No	No
16	No	a, b	b	b	No	b	No	No
17	No	a, b, c, d	a, b, c	a, b	No	a, b	No	No
18	No	b, c, d, e	a, b, c	b	No	b, c	No	No
19	No	a, b, d, f	a, b	a, b, c	No	a, b	No	No
20	No	a, b, e, f	a, b, c	c	No	a, b, c	No	No
21	No	b, c		b	No	b	No	No
22	No	a, b, c, d	a, c	a, b, c	Yes	a, c	No	No
23	No	a, b, c, d, f	a, c	a, c	No	b	No	No
24	No	a, d	b, c	b	No	b	No	No
25	No	b	b	b	No	b, c	No	No
26	No	c		a, b	No	a, b	No	No

*Note*: This table only includes strategies that varied across pilots; strategies used to facilitate process integration are described elsewhere^16^ and were omitted. Only three strategies differentiated pilots that improved cross‐sector collaboration from those that did not (indicated with *). Appendix S2 identifies all collaboration strategies and data sources used to assess each strategy. Appendix S3 includes additional data on pilot characteristics such as urbanicity and populations of focus.

^a^ Contracts include incentives for a, partner engagement; b, data‐sharing infrastructure; c, staffing; d, process goals; e, clinical benchmarks; f, other.

^b^ Data‐sharing infrastructure includes a, access to medical, behavioral health, and social service data; b, real‐time access to shared data for frontline staff; c, event‐based notifications of ED or hospital visits.

^c^ Staffing practices include a, multidisciplinary team includes staff with housing expertise; b, workers with lived experience; c, co‐location of social services staff with medical or behavioral health.

^d^ Client‐centered engagement strategies include a, use of street‐ or shelter‐based outreach to identify or enroll eligible members; b, warm hand‐offs at point of care; c, inclusion of client perspective in design or implementation of WPC.

As a sensitivity analysis, we also analyzed the density and multiplexity of all collaborative relationships, not just those that spanned sectors. Overall, the density and multiplexity of these ties did increase after WPC, with 18 of 26 pilots reporting statistically significant increases in the density or multiplexity of ties. Cross‐case analyses comparing eight pilots that improved both density and multiplexity of ties to eight pilots that did not improve either one yielded similar results to our analysis of factors differentiating pilots that improved cross‐sector collaboration from those that did not. Detailed results are available in Appendix S3.

## 4 DISCUSSION

Health care organizations are increasingly partnering with human services organizations to improve the integration of care for patients with complex medical and social needs. The current study drew on data from a statewide Medicaid demonstration program and applied a conceptual framework of multilevel factors affecting care integration to identify collaboration strategies used by 26 pilots to improve the integration of care. We also assessed factors that differentiated pilots that improved cross‐sector collaboration from those that did not. We identified a wide range of strategies used to promote integration of care. For example, all pilots emphasized the importance of infrastructure development and staffing practices such as the use of multidisciplinary teams (structural integration), data sharing agreements and client consent forms (functional integration), and comprehensive needs assessments and client‐centered care plans (process integration) for effective coordination of care. Similarly, all pilots also described the importance of regular meetings for facilitating partner engagement (normative integration) and improving quality of collaboration between clients and staff at the frontlines of care (interpersonal integration).

However, we found that only three strategies differentiated pilots that improved cross‐sector collaboration from those that did not: collaborative governance, braiding disparate funds to support provision of integrated care, and implementing WPC as part of broader, cross‐sector systems change efforts. Strategies identified as impacting cross‐sector collaboration all focused on facilitating structural or normative integration rather than functional, interpersonal, or process. Structural and normative integration reflect differences in how care is structured and the strength of formal and informal relationships between partners rather than specific actions to improve care coordination for individual clients. For example, siloed, inflexible funding streams have long served as a barrier to cross‐systems alignment.^41^, ^42^ In WPC, the presence of a Section 1115(a) waiver allowing for flexible use of Medicaid funds was viewed as necessary but not sufficient for integration if lead entities did not also adopt collaborative approaches in program design and implementation. Importantly, although all pilots emphasized the importance of developing new data‐sharing infrastructure and agreements, we found these coordination mechanisms were not by themselves sufficient for improved care integration. Prior research on integration has been critiqued for inadequate attention to and measurement of the impact of “social features,” that is, alignment of norms and informal ties^22^, ^31^; our study confirms the importance of these social features to health and human services integration efforts. In WPC, differences in normative integration were perceived as directly impacting effectiveness of strategies focused on improving functional, interpersonal, and process integration, for example, differential partner uptake of data‐sharing systems.

Our study had several limitations that should be considered in interpretation of results. First, we did not interview or survey members, limiting our ability to directly link collaboration strategies to client experience of care. Prior research has demonstrated associations between perceived integration and more distal measures of system effectiveness^43^; however, future research could more directly link the use of identified collaborative practices to client‐rated access or quality of care. Second, although integration is a multidimensional construct, we were only able to measure changes in “macro level” relationships between WPC‐participating entities and not “micro‐level” relationships between specific individuals or groups responsible for care coordination. Qualitative data from our study suggest that differences in inter‐organizational relationships impacted frontline worker behavior; however, future research could more directly assess how changes at one level may impact changes at another.^44^

In 2024, 27 states and the District of Columbia had active or pending Medicaid Section 1115(a) waivers with HRSN provisions, for example, to provide select members with case management, housing services, nutrition support, or employment assistance.^45^ As efforts to integrate health and human services intensify, more information is needed regarding how to maximize the effectiveness of HRSN interventions, and collaboration strategies effective at aligning operations or services across systems. Findings from this study highlight strategies used by cross‐sector partners to facilitate integration of care and confirm the importance of not just data sharing, but collaborative governance practices, braided funding, and investment in upstream systems change to cross‐sector system alignment efforts. Future research could assess the effectiveness of these collaboration strategies in different contexts, and examine their impact on other client or program outcomes.

## Supporting information


**Data S1.** Supporting information.
