# Supplementary material for: Collaboration strategies for bridging health, behavioral health, and social services in California's Medi‐Cal Whole Person Care Pilot Program
Source: Health Serv Res. 2024 Dec 4;60(Suppl 3):e14417. doi: 10.1111/1475-6773.14417 (PMC12052524; doi:10.1111/1475-6773.14417)
Supplement: Supplementary file 1 — Data S1. Supporting information. [file HESR-60-0-s001.docx]

**Supplemental Appendix 1. Characteristics of Medi-Cal Whole Person Care Pilots**

California’s Medi-Cal Whole Person Care (WPC) pilot program was implemented by 25 pilots representing 26 counties and one city in the state. The service area of each pilot typically corresponded to that of a single county. One pilot (Small County Collaborative; SCC) was comprised of two geographically separate rural counties that collaborated on financial claiming and data reporting but designated separate county-specific lead entities and developed distinct partnerships and programs; in the current study, we chose to treat each of these SCC counties as a unique pilot, resulting in a final sample of 26 pilots.

Table 1 provides additional information about each pilot, including the number of partners and a brief description of the pilot.

| **Table 1. Characteristics of WPC Pilots** | | |
| --- | --- | --- |
| **Pilot lead entity** | **# partners (2018)** | **Brief description** |
| Alameda County Health Care Services Agency | 36 | Alameda’s pilot identified eligible members at point of care and using administrative data, connecting them to tiered care management and robust housing support provided by contracted community-based organizations. Major priorities for Alameda’s pilot included the creation of a new shared data system and other infrastructure needed to improve integration of care within the community (e.g., via redesign of the Coordinated Entry system and the behavioral health crisis response system). |
| City of Sacramento | 26 | Sacramento’s pilot was administered by a local consulting firm and focused on individuals experiencing or at-risk of homelessness with the highest service needs and costs. Eligible members were identified using assertive community-based outreach and direct referrals. Enrollees were provided with needed care navigation and expedited access to health, behavioral health, and social services by an interdisciplinary team. All services were delivered via contract with local FQHCs, housing entities, and a local nonprofit that served as the data management entity. |
| Contra Costa Health Services | 12 | Contra Costa’s pilot was a newly developed, stand-alone program, in which predictive risk models were used to identify high utilizers using linked data from multiple sources, including electronic health records from clinics and hospitals, claims, and other county databases. Eligible members were contacted by an “in-house” interdisciplinary team for enrollment and assignment to risk-stratified care management, with field-based services for the highest need. |
| County of Marin Department of Health and Human Services | 30 | Marin’s pilot expanded on and leveraged integration of existing programs and services within the community, such as the Full Services Partnership program, community-based coalitions of homeless service providers, and post-release interventions already being implemented by the probation department. A major focus was on integrating existing efforts by implementing a unified coordinated entry and care management system, promoting bidirectional information sharing between providers, and standardizing screening, assessments, and care coordination activities. |
| County of Orange Health Care Agency | 25 | Orange’s pilot focused on improving existing programs and services for individuals experiencing homelessness, particularly those with SMI. All care coordination services were contracted to the county behavioral health agency and its community partners, and supported by a data sharing platform developed specifically for WPC to facilitate enrollment and care coordination processes, along with real time notifications to the care team and ability to send referrals. WPC funds focused on addressing service gaps, e.g., ensuring closed loop referrals or funding a dedicated social worker for street-based outreach and connection of individuals experiencing homelessness to a medical home. |
| County of San Diego Health and Human Services Agency | 20 | San Diego’s pilot worked primarily with enrollees experiencing homelessness and high utilizers. WPC services included a two-month intensive outreach and engagement phase, followed by formal enrollment with an emphasis on stabilization, maintenance, transition, and aftercare. The pilot utilized tiered care management based on enrollee acuity; services were primarily provided by two contracted community-based providers. Data sharing between the lead entity and participating partners further facilitated the coordination of care, providing field-based access and real-time notifications through a centralized platform branded as ConnectWellSD. |
| County of Santa Cruz Health Services Agency | 19 | Santa Cruz’s pilot focused primarily on improving integration of behavioral health and clinical health within the county. Their pilot emphasized strengthening existing relationships and developing new partnership networks to establish referral pathways for enrollment into WPC and connect enrollees to needed services. Santa Cruz utilized multidisciplinary care coordination teams with a case manager with a social work background as the primary point of contact. The pilot expanded on an existing health information exchange and in 2020, implemented a new electronic case management platform. |
| County of Sonoma Department of Health Services Behavioral Health Division | 14 | Sonoma’s pilot used case managers supported by a larger multidisciplinary team to provide care coordination to individuals experiencing homelessness, at-risk of homelessness, or with SMI/SUD. In implementing WPC, county agencies developed new relationships with local FQHCs centers, with an emphasis on integration of primary care and behavioral health services, and developed a cloud-based mobile care coordination tool with referral capabilities to support data sharing. |
| Kern Medical Center | 16 | Kern’s pilot expanded on a robust care coordination infrastructure already in place within the county healthcare system to improve transitions of care for recently incarcerated individuals and improve access to care for high-utilizers and individuals experiencing homelessness. Eligible members were identified and assessed by a multidisciplinary, clinic-based team and connected to care management (e.g., 90 days enhanced care coordination post-incarceration or care management plus housing support for individuals experiencing homelessness). |
| Kings County Human Services Agency | 9 | Kings’ pilot involved “no wrong door” referrals screened by a contracted, multidisciplinary team that provided case management and referrals to other needed services. A unique attribute of Kings’ pilot was ensuring care plans were finalized with input from all coordinating service providers (e.g., probation officers, social workers, mental health case managers). |
| Los Angeles County Department of Health Services | 51 | Los Angeles’ pilot implemented 16 county-led programs designed for six different populations of focus (e.g., transitions of care for individuals following hospitalization or post-incarceration, SUD navigation and support). Many programs existed prior to WPC but were expanded using WPC funds. Care coordination services were provided by multidisciplinary teams and relied heavily on community health workers supervised by licensed social workers. The pilot also developed a new comprehensive care management platform to facilitate frontline staff workflows and data reporting. |
| Mendocino County Health and Human Services Agency | 9 | Mendocino’s pilot focused on serving individuals with SMI/SUD. All WPC services were contracted out to community providers. The pilot utilized “wellness coaches” with lived experience who spearheaded enrollee outreach, engagement, and care coordination, while also aiding with resource navigation (e.g., grocery drop-offs, benefits applications). “Wellness coaches” were supported by a multidisciplinary team. |
| Monterey County Health Department | 17 | Monterey’s pilot primarily targeted individuals experiencing homelessness, prioritizing individuals with comorbidities and/or high health care utilization. The pilot emphasized field-based outreach at shelters and encampments. Care coordination was provided by a public health nurses and an assistant, typically with a behavioral health background. A key emphasis of the pilot was creating memorandums of understanding with housing developers to establish housing placements; the pilot was also able to secure place-based housing vouchers specifically for WPC enrollees. |
| Napa County | 12 | Napa’s pilot focused on serving individuals experiencing or at-risk of homelessness, and was intended to serve as “the backbone of homeless systems redesign” in the county. The pilot utilized both field- and shelter-based outreach to engage eligible members in WPC, and employed a “Housing First” approach in serving enrollees. Care coordination services were provided by case managers and housing navigators. |
| Placer County Health and Human Services Department | 22 | Placer’s pilot relied primarily on data from the Continuum of Care, the local 2-1-1, and eligible members’ vulnerability scores to prioritize and telephonically enroll members in WPC. Care coordination services were provided by individuals with lived experience similar to that of enrollees, who were supported by a team of nurses, clinicians, and housing specialists. Care coordinators used a collaborative care management platform to track care coordination activities. |
| Riverside University Health System - Behavioral Health | 15 | Riverside’s pilot served individuals on probation with both physical and mental health conditions and who were homeless or at-risk of homelessness. A key focus of the pilot was to support individuals during and after the transition from correctional institutions to the community. RNs co-located in eight probation officers were expected to screen and provide “warm hand-off” referrals of eligible members to community-based RN care managers upon release. |
| San Bernardino County Arrowhead Regional Medical Center | 10 | San Bernardino’s pilot entailed provision of care coordination services by newly developed multidisciplinary teams located at a large, regional medical center. Teams were comprised of patient navigators supported by a nurse, AOD counselor, and social worker. San Bernardino’s pilot emphasized the use of field-based care and held regular “WPC Accountability Review” conferences, where they had dedicated time to discuss every enrollee’s needs and challenges. |
| San Francisco Department of Public Health | 10 | San Francisco’s pilot focused only on individuals experiencing homelessness. Street- and facility-based outreach, coupled with newly developed infrastructure (e.g., navigation centers built using user-centered design), was used to assess enrollees’ health and housing-related needs and engage them in care. Services provided were tiered based on acuity and need. |
| San Joaquin County Health Care Services Agency | 15 | San Joaquin’s pilot relied on street- or shelter-based outreach and referrals to identify eligible members and connect them to care coordination services provided by different entities based on pre-existing relationships and identified need (e.g., individuals with mild mental health needs could receive services from local FQHCs while those with SMI would be connected to county behavioral health). Care plans were shared across all participating entities. The pilot also leveraged a county flexible housing pool (created using non-WPC funds) to address enrollees’ housing needs. |
| San Mateo County Health System | 9 | San Mateo’s pilot focused primarily on expanding existing programs and developing new infrastructure and processes for improving integration of care between historically siloed county departments, e.g., via creation of a new “virtual linkage hub,” data dashboard, and care coordination committee. Eligible members were referred to a triage line and assessed by a nurse who would then connect them to appropriate county programs or resources, e.g., integrated medication assisted treatment team. The primary new intervention developed for WPC was a mobile health clinic team focused on connecting individuals experiencing homelessness to primary care and behavioral health homes. |
| Santa Clara Valley Health and Hospital System | 36 | Santa Clara’s pilot focused on enhancing existing programs and improving integration of care through development of new data sharing infrastructure (e.g., ensuring county custody health and county mental health were using the same electronic health record system). Eligible members were identified using a point system assessing prior use of acute care, with higher values assigned to use of emergency care (psychiatric or medical). Care coordination services were provided within community clinics using standardized care coordination protocols and tiered based on needs and anticipated duration of care. |
| Shasta County Health and Human Services Agency | 11 | Shasta’s pilot focused on serving high utilizers and individuals at risk for or experiencing homelessness. Care teams consisted of multidisciplinary “teamlets,” comprising of case managers, nurses, housing case managers, and various care coordinators. The pilot utilized a “Housing First” approach to care. Partnerships between the lead entity and community clinics were seen as longstanding and identified as facilitating effective coordination of care. |
| SCC: Mariposa Human Services Department | 11 | Mariposa’s pilot primarily targeted high utilizers and individuals with SMI and SUD. Care coordinators were co-situated within the county’s behavioral health, social services, and public health divisions, allowing for tailored enrollee assignments based on need and complexity. Time spent on building trust and rapport prior to enrollment was perceived as critical for assessing prospective enrollees’ motivation to participate, and in facilitating engagement following enrollment. |
| SCC: San Benito County Health and Human Services Agency | 11 | San Benito’s pilot focused on individuals who were at risk for or experiencing homelessness. Enrollees were tiered based on complexity. San Benito emphasized streamlining processes around delivery of housing services and used WPC funds to assist with legal support for issues related to housing/tenancy issues, provided ongoing assistance with enrollee-landlord relationships, and utilized short-term housing options (e.g., shelter or motel stay), where necessary based on enrollee need. |
| Solano County Health and Social Services | 13 | Solano’s pilot prioritized high utilizers and individuals with SMI/SUD. The pilot relied primarily on referrals from medical centers and developed strategic relationships with discharge planners who alerted WPC staff about eligible enrollees. Solano utilized a “Housing First” model and prioritized temporary housing and stabilization to link enrollees to long-term housing. Care coordination services were provided by a contracted community-based provider. |
| Ventura County Health Care Agency | 48 | Ventura’s pilot leveraged a robust, existing “Health Care for the Homeless” program to establish a centralized, electronic care coordination data sharing platform and improve integration of care for individuals experiencing homelessness who were also high utilizers of health care. Field-based care coordination services were provided by a centralized, multidisciplinary team led by a trained community health worker. Ventura also fielded “mobile outreach care pods” to provide services such as immediate access to showers and limited scope medical services. |

AOD=alcohol or other drugs; FQHC=federally qualified health center; RN=registered nurse; SCC=Small County Collaborative; SMI=serious mental illness; SUD=substance use disorder

**Sources**: Number of partners drawn from authors’ analyses of partner rosters and surveys completed by each pilot. Pilot descriptions developed using WPC applications, bi-annual narrative reports, and key informant interviews conducted in 2018-2019 and 2021.

**Supplemental Appendix 2. Data sources**

Table 1 describes all data sources used in this study, and Table 2 summarizes data sources used to assess each collaboration strategy. Table 3 provides example interview questions asked in 2018-2019 and in 2021.

| **Table 1. Overview of Data Sources** | |
| --- | --- |
| **Data Sources** | **Description** |
| ***Materials submitted by WPC pilots to DHCS*** | |
| WPC pilot applications | Applications submitted by pilots to DHCS to participate in WPC, including detailed information on initial participating entities; community need and existing efforts to improve integration of care; pilot governance structure and communication plans; populations of focus; enrollee identification and outreach methodology; WPC services, interventions, care coordination activities, and data sharing; and financing. |
| Narrative reports | Bi-annual progress reports submitted by pilots to DHCS addressing structured topics such as program status, administrative and delivery system infrastructure developed to support achievement of WPC goals, pilot performance on pay-for-outcome metrics or towards earning incentive payments, stakeholder engagement, and successes and challenges experienced with care coordination, data sharing, data collection, and WPC overall. |
| WPC enrollment and utilization reports | Quarterly reports on WPC enrollment and service utilization, 2017-2021 |
| WPC invoices | Annual reports of WPC pilot expenditures by category (infrastructure, services, incentive payments, pay for outcomes or pay for reporting), 2016-2021 |
| ***Primary data collection by the WPC evaluation team*** | |
| LE surveys | Structured surveys administered to WPC LEs in 2018, 2020, and 2021. Each survey addressed different topics (e.g., data sharing, staffing practices, contracting practices, detailed definitions of services provided, use of WPC and non-WPC funds to address client housing needs, care coordination processes, etc.). |
| Partner rosters | Lists of WPC participating entities submitted by each LE in 2018 and 2020 |
| Network surveys | Structured surveys administered to all WPC-participating entities to assess inter-organizational relationships. |
| Key informant interviews | Semi-structured interviews conducted in 2018-2019 and 2021 with organizational leaders, program managers, frontline supervisors, and staff involved in WPC implementation at each pilot. Interview questions varied by participant role and time period. Interview topics are briefly described below, and example interview questions are provided in Table 3.   - In 2018, interviews generally addressed topics such as motivation for participating in WPC, key pilot goals and activities, participation in other initiatives similar to WPC, pre-existing and newly developed data sharing and delivery system infrastructure, care coordination processes, partnerships, client identification and engagement strategies, use of flexible funding strategies (e.g., braided funding, flexible housing pools), critical success factors and lessons learned, and perceived WPC impact. - In 2021, interviews addressed “core elements” of WPC, synergy or overlap with other programs or initiatives in the community, care coordination practices, lessons learned in staffing, lessons learned in coordinating or integrating care for clients, client outreach and engagement strategies; data sharing infrastructure; partner engagement, community engagement in WPC, contracting practices, COVID-19 impact, critical success factors and lessons learned, WPC impact, and WPC sustainability. |

DHCS=California Department of Health Care Services; LE=lead entity; WPC=Whole Person Care

| **Table 2. Data sources used to assess each collaboration strategy** | | | | |
| --- | --- | --- | --- | --- |
|  | **Collaboration strategy** | **Description** | **Data Source(s)** |  |
| **Structural integration** | Collaborative governance | Governing arrangement in which stakeholders are directly engaged in formal decision-making. In this study, we required that the governing arrangement include cross-sector partners, i.e., at least 1 stakeholder from medical, behavioral health, and human services sectors, and that these partners be included in design, planning, and implementation of WPC. | WPC applications which included descriptions of planned governance structures; 2018 and 2020 LE survey; 2018-2019 interviews |  |
|  | Contracts or other legally binding agreements | Contracts, memorandum of understanding, or other legally binding agreements used to formalize inter-agency relationships. In this study, we assessed whether WPC services were directly provided by the LE or contracted out. We also assessed contracting challenges and use of incentive-based payments. | 2018-2019 interviews; 2018 LE survey measures of data sharing agreements (BAAs, MOUs, etc.); 2021 LE survey measures on contracting challenges and whether contracts with WPC partners included incentives for partner engagement, data sharing, staffing, process outcomes, or clinical outcomes. |  |
|  | Infrastructure development | Development of delivery system or data sharing infrastructure needed to improve integration of care, either by addressing gaps in existing systems of care or to improve process integration. | 2018-2019 and 2021 interviews; narrative reports; 2020 LE survey measures of data sharing infrastructure and types of partners that data are shared with |  |
|  | Staffing | Staffing practices perceived as important for integration of care | 2018-2019 and 2021 interviews; 2018 LE and 2020 LE survey measures of staffing practices |  |
|  | Flexible funding | Use of braided funding or other flexible funding strategies to deliver services. Braided funding defined as use of multiple funding streams to support a program or goal, while ensuring funds remain distinct and trackable. | Interviews; narrative reports; 2020 LE survey measures (housing only) |  |
| **Functional integration** | Universal client consent form for release of information | Document securing client’s consent to use, disclose, and exchange protected health information with other designated providers | Interviews; 2020 LE survey measure |  |
|  | Standardized enrollment or referral protocols | Standardized policies and procedures for identifying, enrolling, or referring clients to care | Interviews; narrative reports; 2018 and 2020 LE survey measures regarding use of standardized protocols and care coordination processes |  |
| **Normative integration** | Regular meetings with cross-sector leaders | Regular meetings with cross-sector leaders focused on joint problem-solving, advocacy, or information sharing | Interviews; narrative reports |  |
|  | WPC as part of broader cross-sector systems change effort | Whether WPC was implemented as a standalone program or as part of a broader systems change effort. In this study, we required a focus on cross-sector systems change and for efforts to include organizations from >1 sector | Interviews; WPC applications; 2018 LE survey measure |  |
| **Interpersonal integration** | Client-centered approaches for outreach and engagement | Strategies used to engage clients in care address client-identified needs and priorities. | 2018-2019 and 2021 interviews; narrative reports; 2020 and 2021 LE survey measures regarding client outreach and engagement strategies |  |
|  | Use of staff with lived experience | Use of community health workers, peer support specialists, or other staff with lived experience as a strategy for improving quality of collaboration with clients | 2018-2019 interviews; 2020 LE survey measures |  |
|  | Staff training | Staff training needed to implement client-centered approaches or ensure staff understanding of expected care coordination activities | 2018-2019 interviews; narrative reports; 2018 LE survey measures |  |
|  | Regular case conferences, huddles, or other team meetings | Regular meetings involving frontline staff and other stakeholders from different sectors to discuss client care | Interviews; narrative reports; 2018 LE survey measures |  |
| **Process integration** | Comprehensive needs assessment using pre-specified, structured tool | Tools used to facilitate comprehensive assessment of client’s physical health, behavioral health, and social needs. | 2018 and 2020 LE survey measures of care coordination processes and of specific needs assessments tools used |  |
|  | Client-centered care plan accessible to all staff responsible for care coordination | Whether care coordination activities were informed by a client-centered care plan, and whether this care plan was accessible to all staff responsible for care coordination | Interviews; 2018 and 2020 LE survey measures of care coordination processes |  |

LE=Lead entity; WPC=Whole Person Care

| **Table 3. Example Interview Domains and Questions*** | |
| --- | --- |
| **Interview Domain** | **Example Interview Questions** |
| **Motivation for WPC and broader systems change goals** | - Can you tell me a little bit about your organization’s primary motivation for participating in WPC? - How does WPC fit with your organization’s overall strategic priorities? - Please tell us about synergy or potential overlap of WPC with any other programs or initiatives in your community. - Was your WPC pilot implemented as a standalone program, an expansion of existing program(s), or as part of broader systems change efforts? |
| **Pilot characteristics** | - Can you tell me about how your WPC pilot was first developed? How did you decide who to partner with on this initiative? What factors affected your decision to focus on specific populations or services? - What do you view as the “core elements” of your pilot, in terms of partnership, infrastructure, or services developed and delivered to improve integration of care? - [For frontline staff] What do you feel is innovative about WPC, either in terms of the role it fits in your community or the work you do with clients? |
| **Infrastructure** | - What new data sharing infrastructure was developed as a result of your participation in WPC? What about delivery system infrastructure? What infrastructure was needed to improve coordination or integration of care within your community? - [For frontline staff] What type of information is currently being collected about clients? How helpful do you find available information for informing your work with clients? What about for coordinating with other providers or understanding what these providers are doing? What, if anything, would you change about the way your organization tracks information about your clients? |
| **Care coordination and integration of care** | - What does care coordination “look like” in your pilot? Who are the staff involved? How are responsibilities distributed across the team? What data sharing infrastructure was developed to facilitate care coordination? Are there any standard referral protocols or pathways in place that staff are asked to follow? - Can you speak to any major lessons learned in integrating care for WPC members? \ - [For frontline staff] How would you describe your job to someone who knew nothing about it? How do you communicate or coordinate care with other providers outside your organization / in the community? |
| **Partnerships** | - Please tell us about partnerships developed as a result of WPC. How did you decide which partnerships to pursue? Which partners did you feel were most critical to the success of WPC? - What challenges have you encountered in coordinating WPC activities with partner organizations? What strategies have you found helpful at breaking down siloes between partners? - Have you noticed any significant changes in partner relationships or ways of working together as a result of WPC? Why or why not? Can you provide an example? - We previously asked LEs to identify partner organizations. We saw you had a total of X partners in [year]. Is that still accurate? Were there any other organizations involved in WPC – e.g., “unfunded” partners that didn’t directly receive funds but were still important for successful design, implementation, or impact? |
| **Member outreach and engagement** | - What is your process for identifying and engaging eligible members in WPC? - What strategies have you found most effective? What has been most challenging about this process? - [For frontline staff] How do clients get connected to WPC? What strategies have you found most helpful for identifying and engaging clients? |
| **Funding** | - Has your organization been able to use WPC to leverage additional funding or resources? - We noticed in your [application / narrative report], that you are using [X innovative funding mechanism]. Can you tell us a little bit about how this works and how it came about? - One challenge we have heard is that WPC funds cannot be used to directly pay for housing and WPC services and activities cannot duplicate services already available in Medicaid. How has your pilot addressed this challenge? |
| **Lessons learned** | - What do you view as critical success factors affecting whether WPC goals are realized (e.g., partnerships, infrastructures, types of services provided, staff used, etc.)? - Do you have any advice for other counties or states considering whether to adopt similar programs? - [For frontline staff] What are your perceptions of the overall impact and value of WPC in your community? How does WPC compare to other programs you have worked on/ for? If you could change one thing about WPC, what would it be? |

* This is not a comprehensive list of interview domains or questions, but provides examples of interview domains and questions salient to this study.

**Supplemental Appendix 3. Changes in collaboration before and after WPC**

Table 1 provides results for individual, pilot-level changes in density or multiplexity of cross-sector ties before and after WPC.

| **Table 1. Select pilot-level characteristics and cross-sector collaboration before and after WPC** | | | | | | | | |
| --- | --- | --- | --- | --- | --- | --- | --- | --- |
| **Pilot** | **Urbanicity** | **Populations of focus^a^** | **Density: Any tie** | | **Multiplexity** | | **Improved cross-sector collaboration?** |  |
|  |  |  | **Prior to WPC** | **After WPC** | **Prior to WPC** | **After WPC** |  |  |
| 1 | Urban | 1,2 | 0.34 | 0.45* | 1.96 | 2.04 | Yes |  |
| 2 | Rural | 3,4 | 0.75 | 0.81 | 4.05 | 4.22 | Yes^+^ |  |
| 3 | Urban | 1,2,3,4,5 | 0.23 | 0.33* | 1.48 | 2.03* | Yes |  |
| 4 | Suburban | 2 | 0.47 | 0.54 | 1.89 | 2.76* | Yes |  |
| 5 | Suburban | 2 | 0.52 | 0.79* | 2.21 | 2.76 | Yes |  |
| 6 | Urban | 2,4 | 0.29 | 0.32 | 1.83 | 2.66* | Yes |  |
| 7 | Urban | 1,2 | 0.61 | 0.77* | 2.08 | 2.15 | Yes |  |
| 8 | Urban | 1,2,4 | 0.56 | 0.71* | 2.17 | 2.5 | Yes |  |
| 9 | Urban | 1 | 0.42 | 0.62* | 3.91 | 3.07 | Yes |  |
| 10 | Suburban | 1 | 0.56 | 0.54 | 1.6 | 2.62* | Yes |  |
| 11 | Urban | 1 | 0.44 | 0.48 | 1.84 | 2.11* | Yes |  |
| 12 | Urban | 1 | 0.42 | 0.47 | 2.1 | 2.07 | No |  |
| 13 | Suburban | 1,2,5 | 0.48 | 0.52 | 2.06 | 2.05 | No |  |
| 14 | Suburban | 1,2 | 0.51 | 0.52 | 1.92 | 2.14 | No |  |
| 15 | Rural | 1,4 | 0.63 | 0.63 | 2.27 | 2.9 | No |  |
| 16 | Rural | 4 | 0.56 | 0.57 | 2.56 | 2.86 | No |  |
| 17 | Suburban | 1,2,3,4,5 | 0.44 | 0.44 | 2.12 | 2.06 | No |  |
| 18 | Urban | 5 | 0.66 | 0.66 | 1.39 | 1.35 | No |  |
| 19 | Urban | 1,2 | 0.50 | 0.56 | 1.64 | 2.05 | No |  |
| 20 | Rural | 1,2 | 0.58 | 0.42 | 2.32 | 2.24 | No |  |
| 21 | Urban | 1 | 0.64 | 0.64 | 2.87 | 2.71 | No |  |
| 22 | Urban | 2 | 0.67 | 0.65 | 2.68 | 2.84 | No |  |
| 23 | Urban | 1 | 0.39 | 0.41 | 1.88 | 2.07 | No |  |
| 24 | Suburban | 3,4 | 0.55 | 0.57 | 2.24 | 2.29 | No |  |
| 25 | Suburban | 1,4 | 0.69 | 0.76 | 2.39 | 2.7 | No |  |
| 26 | Suburban | 2,4 | 0.43 | 0.46 | 2.43 | 3.03 | No |  |

**Source:** Authors’ analyses of roster-based network surveys administered to all WPC-participating entities assessing collaborative relationships before and after implementation of WPC. This table only reports on cross-sector relationships. Urbanicity based on data provided by the 2020 California State Association of Counties.

^a^ Populations of focus: 1=High utilizer, 2=Homeless or at-risk of homelessness, 3=Chronic physical conditions, 4=SMI/SUD, 5=Justice-involved

*Significant changes based on results of paired t-tests (p<0.05).

^+^Although increases in density or multiplexity of cross-sector ties were not statistically significant for pilot 2, baseline density and multiplexity were so high that we considered this pilot as improving cross-sector collaboration in our cross-case analyses and identified as “Yes” in this table.

***Sensitivity analysis: Changes in overall collaboration before and after WPC***

As a sensitivity analysis, we also assessed collaboration based on average network density and multiplexity of ties between all WPC partners, not just cross-sector partners. As shown in Table 2, prior to WPC, average network density was 0.54 (range 0.21 – 0.92), meaning that on average, over half of organizations (54%) had collaborative relationships prior to WPC. The most common type of collaborative relationship between partners was client referrals (mean 0.35), followed by joint planning or advocacy (mean 0.28), and communication about client needs or care (mean 0.27). The least common collaborative relationships were data sharing (mean 0.20) and joint service delivery (mean 0.16). By the end of 2018, approximately two years after WPC implementation, average network density increased (mean 0.59; range 0.27 – 0.97). While client referrals remained the most common type of collaborative relationship (mean 0.38), increases in density of referrals were not significant at the p<0.05 level; however, increases in density of all other types of collaborative relationships were statistically significant. Multiplexity of ties also increased from an average of 1.95 to 3.05, meaning that on average, partners worked together in more ways than they had prior to WPC; these changes were also statistically significant.

**Table 2. Changes in collaboration before and after WPC**

|  | **Prior to WPC** | | **After WPC (2018)** | |
| --- | --- | --- | --- | --- |
|  | **Mean (S.D.)** | **Range** | **Mean (S.D.)** | **Range** |
| Network density: Any tie | 0.54 (0.18) | 0.21 – 0.92 | 0.59 (0.27)* | 0.27 – 0.97 |
| Joint advocacy or planning | 0.28 (0.13) | 0.06 – 0.67 | 0.36 (0.17)* | 0.07 – 0.89 |
| Data sharing | 0.20 (0.13) | 0.04 – 0.61 | 0.28 (0.16)* | 0.05 – 0.72 |
| Client referrals | 0.35 (0.16) | 0.10 – 0.78 | 0.38 (0.18) | 0.14 – 0.83 |
| Communication about client needs or care | 0.27 (0.15) | 0.10 – 0.72 | 0.34 (0.17)* | 0.14 – 0.81 |
| Joint service delivery | 0.16 (0.15) | 0.02 – 0.67 | 0.20 (0.17)* | 0.03 – 0.67 |
| Multiplexity of ties | 1.95 (0.95) | 0.1 – 4.05 | 3.05 (1.03)* | 0.78 – 4.82 |

**Source:** Authors’ analyses of roster-based network surveys administered to all WPC-participating entities assessing changes in collaborative relationships before and after implementation of WPC

*Significant change after WPC, p<0.05

Analysis of individual pilot-level networks indicated that 18 pilots reported statistically significant increases in density or multiplexity of ties following WPC (p<0.05), and 8 pilots did not increase either density or multiplexity of ties. Of the 18 pilots that reported statistically significant increases in density or multiplexity of ties following WPC, 8 improved both density and multiplexity of ties. As a sensitivity analysis, we repeated our cross-case analyses, but focused on comparing the 8 pilots that increased both density and multiplexity of ties to the 8 pilots that did not improve either.

**Table 3. Select collaboration strategies used by pilots to facilitate integration of care^+^**

| Pilot | Collaborative governance* | Contract incentives^1^ | Data sharing^2^ | Staffing practices^3^ | Braided funding* | Client engagement^4^ | WPC as part of broader systems change* | Did pilot improve density or multiplexity? |
| --- | --- | --- | --- | --- | --- | --- | --- | --- |
| 1 | Yes | b, c | b, c | a, b | Yes | c | Yes | Both |
| 2 | Yes | a, c, d, e | a, c | a, b, c | Yes | a, b | Yes | Both |
| 3 | Yes | a, b, c | a | b, c | Yes | a, b | Yes | Both |
| 4 | Yes | c, d, e | a | a | Yes | a, b, c | Yes | Both |
| 5 | Yes | b, c | a, b, c | b, c | Yes | a, b, c | Yes | Both |
| 6 | Yes | f | a, b | a, b, c | Yes | a, b, c | Yes | Both |
| 7 | Yes | b, d | c |  | Yes | a, b | Yes | Both |
| 8 | Yes | c, e |  | a, b | Yes | a, b, c | Yes | Both |
| 9 | No | a, b ,c, d, e | c | c | No | a, b, c | No | Neither |
| 10 | No | b, c |  | a, b, c | No | a, b, c | No | Neither |
| 11 | No | a, b, c, d | a, b, c | a, b, c | No | c | No | Neither |
| 12 | No | b, c, d, e | a, c | c | No | a, b, c | No | Neither |
| 13 | No | a, b, c, e, f | c | b, c | Yes | a, c | No | Neither |
| 14 | No | b, f | a, c | b | No |  | No | Neither |
| 15 | No | a, b, c, |  | a | No | a, b, c | No | Neither |
| 16 | No | b, c, d, e | a | a, b | No | a, b, c | No | Neither |

^+^ This table only reports data on the 16 pilots included in cross-case comparisons, i.e., eight pilots that improved density and multiplexity of ties compared to eight pilots that did not improve either. Only select pilot characteristics are shown in the table. Collaboration strategies that differentiated pilots that improved collaboration from those that did not are indicated with *. To limit risk of deductive disclosure, pilot numbers shown in this table are randomly assigned and do not match pilot numbers shown in the manuscript, in Appendix S1, or in other Appendix S2 tables.

^1^ Contracts include incentives for a = partner engagement, b= data sharing infrastructure, c= staffing, d=process goals, e=clinical benchmarks, f=other

^2^ Data sharing infrastructure includes a=access to medical, behavioral health, and social service data, b=real-time access to shared data for frontline staff, c=event-based notifications of ED or hospital visits

^3^ Staffing practices include a=multidisciplinary team includes staff with housing expertise, b=workers with lived experience, c=co-location of social services staff with medical or behavioral health

^4^ Client engagement strategies include a=use of street- or shelter-based outreach to identify or enroll eligible members, b=warm hand-offs at point of care, c=inclusion of client perspective in design or implementation of WPC
